# 1-(3,5-Dimeth­oxy­benz­yl)-1*H*-pyrrole

**DOI:** 10.1107/S1600536812015024

**Published:** 2012-04-13

**Authors:** Yueqing Li, Xu Zhang, Shiyong Huo, Wei Huang, Weijie Zhao

**Affiliations:** a School of Pharmaceutical Science and Technology, Dalian Unversity of Technology, PO Box G303, Linggong Road 2, Dalian 116024, People’s Republic of China

## Abstract

The title compound, C_13_H_15_NO_2_, was synthesized from 3,5-dimeth­oxy­benzaldehyde. The dihedral angle between the pyrrole and benzene rings is 89.91 (5)°. In the crystal, weak C—H⋯O and C—H⋯π interactions link the mol­ecules into a three-dimensional network.

## Related literature

For the anti-HIV-1 activity of *N*-(aryl­meth­yl)-pyrrole, see: Liu *et al.* (2008 ▶); Teixeira *et al.* (2008 ▶). For a related structure, see: Wang *et al.* (2011 ▶). For the synthesis of 3,5-dimeth­oxy-benzyl­amine, see: Yraola *et al.* (2006 ▶).
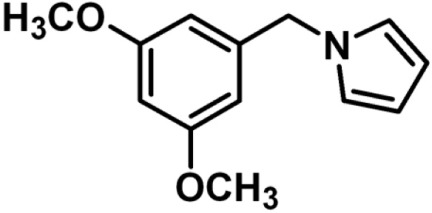


## Experimental

### 

#### Crystal data

C_13_H_15_NO_2_
*M* *_r_* = 217.26
Monoclinic, 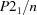

*a* = 9.7569 (11) Å
*b* = 12.2303 (10) Å
*c* = 10.4181 (10) Å
β = 113.720 (7)°
*V* = 1138.2 (2) Å^3^
*Z* = 4
Mo *K*α radiation
μ = 0.09 mm^−1^
*T* = 153 K
0.21 × 0.21 × 0.16 mm

#### Data collection

Bruker APEXII CCD diffractometer
7643 measured reflections
2230 independent reflections
1717 reflections with *I* > 2σ(*I*)
*R*_int_ = 0.027

#### Refinement

*R*[*F* ^2^ > 2σ(*F* ^2^)] = 0.041
*wR*(*F* ^2^) = 0.140
*S* = 0.99
2230 reflections
145 parameters
H-atom parameters constrained
Δρ_max_ = 0.15 e Å^−3^
Δρ_min_ = −0.18 e Å^−3^

### 

Data collection: *APEX2* (Bruker, 2005 ▶); cell refinement: *SAINT-Plus* (Bruker, 2001 ▶); data reduction: *SAINT-Plus*; program(s) used to solve structure: *SHELXS97* (Sheldrick, 2008 ▶); program(s) used to refine structure: *SHELXL97* (Sheldrick, 2008 ▶); molecular graphics: *SHELXTL* (Sheldrick, 2008 ▶); software used to prepare material for publication: *SHELXTL*.

## Supplementary Material

Crystal structure: contains datablock(s) I, global. DOI: 10.1107/S1600536812015024/rk2345sup1.cif

Structure factors: contains datablock(s) I. DOI: 10.1107/S1600536812015024/rk2345Isup2.hkl

Supplementary material file. DOI: 10.1107/S1600536812015024/rk2345Isup3.cml

Additional supplementary materials:  crystallographic information; 3D view; checkCIF report

## Figures and Tables

**Table 1:** Hydrogen-bond geometry (Å, °) *Cg* is the centroid of the C6–C11 ring.

*D*—H⋯*A*	*D*—H	H⋯*A*	*D*⋯*A*	*D*—H⋯*A*
C1—H1*A*⋯*Cg*^i^	0.93	2.79	3.694 (2)	165
C2—H2*A*⋯O2^ii^	0.93	2.72	3.527 (2)	146
C5—H5*A*⋯O2^iii^	0.97	2.68	3.609 (2)	161

Symmetry codes: (i) 
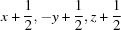
; (ii) 
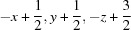
; (iii) 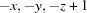.

**Fig. 1.**
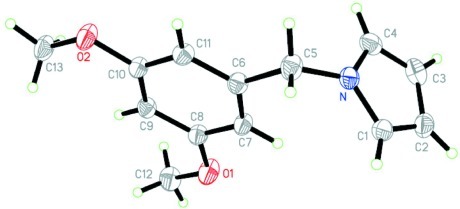
The molecular structure of title compound, showing the atom numbering scheme. Displacement ellipsoids are drawn at 30% probability level. H atoms are presented as a small spheres of arbitrary radius.

**Fig. 2.**
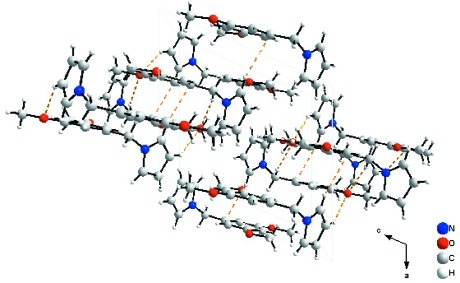
A view of the packing of the title compound along b axis.

**Fig. 3.**
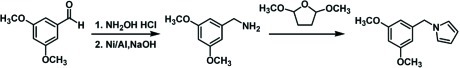
The synthetic route of the title compound.

**Fig. 4.**
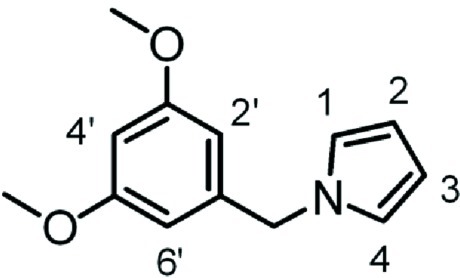
The structure of title compound, with atoms labeling corresponding to the characterization by NMR.

## supplementary crystallographic information

### Comment

A lot of *N*-(arylmethyl)-pyrrole derivatives show anti-HIV-1 activities, such as inhibitory activity on *gp*41 six-helix bundle formation in both molecule modeling study (Teixeira *et al.*, 2008) and activity assay (Liu *et al.*, 2008). The title compound may possess the same qualities. The title compound is prepared *via* two steps and the product of the first step can be added to the solution of the second step without purification.

In the title compound, as shown in Fig. 1, the pyrrole and benzene rings are on the different plane. The dihedral angle between the two plane is 89.91 (5)° and close to the dihedral angle in 1-benzyl-*N*-methyl-1*H*-pyrrole-2-carboxamide (Wang *et al.*, 2011). The N-C5-C6-C7 torsion angle is 26.37 (20)°. The structure is stabilized by the non-classical hydogen bonds (Table 1). The packing diagram is presented in Fig. 2.

### Experimental

Starting material is 3,5-dimethoxy-benzaldehyde (20.9 g, 126 mmol) (Fig. 3). For the first step, 3,5-dimethoxy-benzylamine is prepared according to (Yraola *et al.*, 2006). 2,5-Dimethoxytetrahydrofuran (14.2 g, 108 mmol) and glacial acetic acid (150 ml) were added to the first step product. After stirring at 333 K for 6 h, solvent was removed under reduced pressure. The crude product was purified by flash column chromatography (petrol ether / *Et*O*Ac* (10 / 1), yielding the title compound (0.98 g, 52%) as a white solid. The product (16 mg) was dissolved in ethyl ether (1 ml) and methanol (0.05 ml). Single crystals suitable for X-ray diffraction experiment was obtained from the solution by cooling at 273 K for seven days. The molecule was characterized by NMR (Fig. 4).

^1^H NMR (400 MHz, CDCl_3_): δ 6.68(t, *J* = 2.1 Hz, 2H, H-2, H-5), 6.36(t, *J* = 2.2 Hz, 1H, H-4'), 6.25(d, *J* = 2.2 Hz, 2H, H-2', H-6'), 6.18(t, *J* = 2.1 Hz, 2H, H-3, H-4), 4.99(s, 2H, CH_2_), 3.73(s, 6H, OCH_3_). ^13^C NMR (100 MHz, CDCl_3_): δ 161.2, 140.7, 121.3, 108.6, 105.07, 99.4, 55.3, 53.4. HRMS (ES^+^): *M*/*z* [*M*+Na]^+^ calcd. for C_13_H_15_NO_2_Na: 240.1001; found: 240.1006.

### Refinement

All H atoms attached to C atoms were treated as riding, withC–H = 0.96Å for methyl group, C–H = 0.97Å for methylene group, and C–H = 0.93Å for aromatic ring, with *U*_iso_(H) = 1.2*U*_eq_(C) of the carrier atoms to which they are attached and *U*_iso_(H) = 1.5*U*_eq_(C) for the methyl groups.

### Crystal data

**Table:**

C_13_H_15_NO_2_	*F*(000) = 464
*M**_r_* = 217.26	*D*_x_ = 1.268 Mg m^−^^3^
Monoclinic, *P*2_1_/*n*	Mo *K*α radiation, λ = 0.71073 Å
Hall symbol: -P 2yn	Cell parameters from 2351 reflections
*a* = 9.7569 (11) Å	θ = 2.8–26.0°
*b* = 12.2303 (10) Å	µ = 0.09 mm^−^^1^
*c* = 10.4181 (10) Å	*T* = 153 K
β = 113.720 (7)°	Needle, colorless
*V* = 1138.2 (2) Å^3^	0.21 × 0.21 × 0.16 mm
*Z* = 4	

### Data collection

**Table:**

Bruker APEXII CCD diffractometer	1717 reflections with *I* > 2σ(*I*)
Radiation source: fine-focus sealed tube	*R*_int_ = 0.027
Graphite monochromator	θ_max_ = 26.0°, θ_min_ = 2.7°
φ and ω scans	*h* = −12→8
7643 measured reflections	*k* = −15→15
2230 independent reflections	*l* = −11→12

### Refinement

**Table:**

Refinement on *F*^2^	Primary atom site location: structure-invariant direct methods
Least-squares matrix: full	Secondary atom site location: difference Fourier map
*R*[*F*^2^ > 2σ(*F*^2^)] = 0.041	Hydrogen site location: inferred from neighbouring sites
*wR*(*F*^2^) = 0.140	H-atom parameters constrained
*S* = 0.99	*w* = 1/[σ^2^(*F*_o_^2^) + (0.1*P*)^2^] where *P* = (*F*_o_^2^ + 2*F*_c_^2^)/3
2230 reflections	(Δ/σ)_max_ = 0.014
145 parameters	Δρ_max_ = 0.15 e Å^−^^3^
0 restraints	Δρ_min_ = −0.18 e Å^−^^3^

### Special details

**Table:**

Geometry. All s.u.'s (except the s.u. in the dihedral angle between two l.s. planes) are estimated using the full covariance matrix. The cell s.u.'s are taken into account individually in the estimation of s.u.'s in distances, angles and torsion angles; correlations between s.u.'s in cell parameters are only used when they are defined by crystal symmetry. An approximate (isotropic) treatment of cell s.u.'s is used for estimating s.u.'s involving l.s. planes.
Refinement. Refinement of *F*^2^ against ALL reflections. The weighted *R*-factor *wR* and goodness of fit *S* are based on *F*^2^, conventional *R*-factors *R* are based on *F*, with *F* set to zero for negative *F*^2^. The threshold expression of *F*^2^ > σ(*F*^2^) is used only for calculating *R*-factors(gt) *etc*. and is not relevant to the choice of reflections for refinement. *R*-factors based on *F*^2^ are statistically about twice as large as those based on *F*, and *R*-factors based on ALL data will be even larger.

### Fractional atomic coordinates and isotropic or equivalent isotropic displacement parameters (Å^2^)

**Table:**

	*x*	*y*	*z*	*U*_iso_*/*U*_eq_	
N	0.14877 (13)	0.21993 (9)	0.87889 (11)	0.0396 (3)	
O1	0.13871 (14)	0.44438 (8)	0.47582 (11)	0.0572 (4)	
O2	0.08702 (13)	0.07035 (8)	0.30839 (10)	0.0536 (3)	
C11	0.13905 (15)	0.11206 (11)	0.54195 (14)	0.0397 (4)	
H11A	0.1407	0.0373	0.5591	0.048*	
C7	0.16360 (16)	0.29689 (11)	0.62375 (14)	0.0407 (4)	
H7A	0.1814	0.3469	0.6959	0.049*	
C9	0.10819 (16)	0.26083 (11)	0.37960 (14)	0.0384 (4)	
H9A	0.0884	0.2858	0.2895	0.046*	
C5	0.19169 (18)	0.14323 (12)	0.79460 (15)	0.0478 (4)	
H5A	0.1358	0.0759	0.7850	0.057*	
H5B	0.2971	0.1261	0.8437	0.057*	
C8	0.13589 (16)	0.33349 (11)	0.48959 (14)	0.0394 (4)	
C10	0.11093 (15)	0.14936 (10)	0.40823 (14)	0.0381 (4)	
C1	0.24214 (17)	0.29419 (12)	0.96984 (15)	0.0455 (4)	
H1A	0.3453	0.2980	0.9977	0.055*	
C6	0.16472 (15)	0.18558 (11)	0.65030 (14)	0.0380 (4)	
C12	0.0972 (2)	0.48874 (13)	0.33928 (16)	0.0573 (5)	
H12A	0.1015	0.5671	0.3448	0.086*	
H12B	−0.0028	0.4662	0.2808	0.086*	
H12C	0.1650	0.4629	0.3000	0.086*	
C4	0.00631 (16)	0.24161 (13)	0.86324 (15)	0.0475 (4)	
H4A	−0.0788	0.2035	0.8059	0.057*	
C2	0.15797 (19)	0.36182 (13)	1.01276 (16)	0.0514 (4)	
H2A	0.1932	0.4197	1.0754	0.062*	
C3	0.00937 (18)	0.32851 (14)	0.94572 (16)	0.0528 (4)	
H3A	−0.0726	0.3599	0.9556	0.063*	
C13	0.0520 (2)	0.10539 (13)	0.16790 (15)	0.0537 (4)	
H13A	0.0360	0.0427	0.1082	0.081*	
H13B	0.1336	0.1478	0.1651	0.081*	
H13C	−0.0370	0.1493	0.1359	0.081*	

### Atomic displacement parameters (Å^2^)

**Table:**

	*U*^11^	*U*^22^	*U*^33^	*U*^12^	*U*^13^	*U*^23^
N	0.0424 (7)	0.0461 (7)	0.0284 (6)	−0.0016 (5)	0.0122 (5)	0.0034 (5)
O1	0.0951 (9)	0.0343 (6)	0.0415 (6)	−0.0005 (5)	0.0266 (6)	0.0034 (4)
O2	0.0836 (8)	0.0395 (6)	0.0364 (6)	−0.0022 (5)	0.0230 (6)	−0.0046 (4)
C11	0.0453 (8)	0.0327 (7)	0.0388 (8)	0.0017 (6)	0.0146 (7)	0.0029 (6)
C7	0.0502 (9)	0.0380 (8)	0.0321 (8)	−0.0007 (6)	0.0146 (6)	−0.0032 (6)
C9	0.0433 (8)	0.0409 (8)	0.0304 (7)	0.0010 (6)	0.0142 (6)	0.0035 (6)
C5	0.0616 (10)	0.0434 (8)	0.0366 (8)	0.0042 (7)	0.0180 (7)	0.0038 (6)
C8	0.0473 (8)	0.0329 (7)	0.0387 (8)	0.0005 (6)	0.0180 (6)	0.0012 (6)
C10	0.0433 (8)	0.0362 (7)	0.0343 (7)	0.0005 (6)	0.0150 (6)	−0.0028 (6)
C1	0.0442 (8)	0.0564 (9)	0.0331 (8)	−0.0090 (7)	0.0126 (6)	0.0010 (7)
C6	0.0401 (8)	0.0393 (8)	0.0326 (7)	0.0021 (6)	0.0126 (6)	0.0025 (6)
C12	0.0857 (12)	0.0398 (8)	0.0516 (10)	0.0030 (8)	0.0332 (9)	0.0121 (7)
C4	0.0390 (8)	0.0630 (10)	0.0367 (8)	−0.0048 (7)	0.0114 (7)	0.0107 (7)
C2	0.0703 (11)	0.0494 (9)	0.0366 (8)	−0.0056 (8)	0.0237 (8)	−0.0016 (7)
C3	0.0556 (10)	0.0625 (10)	0.0472 (9)	0.0140 (8)	0.0278 (8)	0.0130 (8)
C13	0.0745 (11)	0.0525 (9)	0.0329 (8)	−0.0035 (8)	0.0203 (8)	−0.0068 (7)

### Geometric parameters (Å, º)

**Table:**

N—C4	1.3589 (19)	C5—C6	1.5097 (19)
N—C1	1.3631 (18)	C5—H5A	0.9700
N—C5	1.4568 (18)	C5—H5B	0.9700
O1—C8	1.3652 (16)	C1—C2	1.362 (2)
O1—C12	1.4207 (17)	C1—H1A	0.9300
O2—C10	1.3693 (16)	C12—H12A	0.9600
O2—C13	1.4284 (17)	C12—H12B	0.9600
C11—C6	1.3849 (19)	C12—H12C	0.9600
C11—C10	1.3845 (19)	C4—C3	1.359 (2)
C11—H11A	0.9300	C4—H4A	0.9300
C7—C6	1.3883 (19)	C2—C3	1.393 (2)
C7—C8	1.3868 (19)	C2—H2A	0.9300
C7—H7A	0.9300	C3—H3A	0.9300
C9—C8	1.3880 (19)	C13—H13A	0.9600
C9—C10	1.3934 (19)	C13—H13B	0.9600
C9—H9A	0.9300	C13—H13C	0.9600
			
C4—N—C1	108.58 (13)	C2—C1—H1A	126.0
C4—N—C5	125.56 (13)	N—C1—H1A	126.0
C1—N—C5	125.05 (13)	C11—C6—C7	119.33 (13)
C8—O1—C12	118.35 (12)	C11—C6—C5	119.39 (12)
C10—O2—C13	117.65 (11)	C7—C6—C5	121.27 (13)
C6—C11—C10	120.26 (12)	O1—C12—H12A	109.5
C6—C11—H11A	119.9	O1—C12—H12B	109.5
C10—C11—H11A	119.9	H12A—C12—H12B	109.5
C6—C7—C8	120.00 (13)	O1—C12—H12C	109.5
C6—C7—H7A	120.0	H12A—C12—H12C	109.5
C8—C7—H7A	120.0	H12B—C12—H12C	109.5
C8—C9—C10	117.98 (12)	N—C4—C3	108.38 (13)
C8—C9—H9A	121.0	N—C4—H4A	125.8
C10—C9—H9A	121.0	C3—C4—H4A	125.8
N—C5—C6	113.71 (12)	C1—C2—C3	107.53 (15)
N—C5—H5A	108.8	C1—C2—H2A	126.2
C6—C5—H5A	108.8	C3—C2—H2A	126.2
N—C5—H5B	108.8	C4—C3—C2	107.42 (14)
C6—C5—H5B	108.8	C4—C3—H3A	126.3
H5A—C5—H5B	107.7	C2—C3—H3A	126.3
O1—C8—C7	115.00 (13)	O2—C13—H13A	109.5
O1—C8—C9	123.69 (13)	O2—C13—H13B	109.5
C7—C8—C9	121.31 (13)	H13A—C13—H13B	109.5
O2—C10—C11	115.85 (12)	O2—C13—H13C	109.5
O2—C10—C9	123.04 (12)	H13A—C13—H13C	109.5
C11—C10—C9	121.11 (13)	H13B—C13—H13C	109.5
C2—C1—N	108.07 (14)		

### Hydrogen-bond geometry (Å, º)

Cg is the centroid of the C6–C11 ring.

**Table:**

*D*—H···*A*	*D*—H	H···*A*	*D*···*A*	*D*—H···*A*
C1—H1*A*···*Cg*^i^	0.93	2.79	3.6935 (19)	165
C2—H2*A*···O2^ii^	0.93	2.72	3.527 (2)	146
C5—H5*A*···O2^iii^	0.97	2.68	3.609 (2)	161

Symmetry codes: (i) *x*+1/2, −*y*+1/2, *z*+1/2; (ii) −*x*+1/2, *y*+1/2, −*z*+3/2; (iii) −*x*, −*y*, −*z*+1.
